# A Novel Internal Electrocautery‐Assisted Lid‐Tightening Maneuver in Transconjunctival Lower Blepharoplasty: Case Series and Outcomes

**DOI:** 10.1002/ccr3.72177

**Published:** 2026-02-25

**Authors:** Afsaneh Sadeghzadeh Bazargan, Alireza Jafarzadeh, Roya Zeinali

**Affiliations:** ^1^ Department of Dermatology Hazrat Fatemeh Hospital, School of Medicine, Iran University of Medical Sciences Tehran Iran

**Keywords:** case series, electrocautery‐assisted lid tightening, eyelid malposition prevention, lower eyelid rejuvenation, transconjunctival blepharoplasty

## Abstract

The internal electrocautery‐assisted lid‐tightening maneuver, combined with transconjunctival blepharoplasty, offers a scarless and effective approach to improve lower eyelid contour and stability, minimizing the risk of postoperative malposition.

## 1 Introduction

Lower eyelid blepharoplasty is one of the most commonly performed procedures in facial rejuvenation, aiming to correct dermatochalasis, fat prolapse, and age‐related lid laxity [1]. The transconjunctival approach has gained popularity because it avoids external incisions and visible scarring, while providing access to herniated orbital fat [2]. However, postoperative complications such as lid malposition, ectropion, and retraction remain a concern, particularly in patients with borderline eyelid laxity [3].

Several adjunctive techniques have been introduced to improve lid tone and reduce the risk of malposition, ranging from lateral canthopexy to laser‐assisted tightening procedures [4, 5]. Despite their benefits, these methods may require additional dissection, sutures, or specialized equipment, which can increase operative time and morbidity [6].

In this context, a simple and minimally invasive maneuver that enhances eyelid stability without external scars would be of great clinical value. We describe a novel internal lid‐tightening technique, in which the conjunctiva was incised to allow fat removal, while the actual procedure is performed on the underlying plane, specifically targeting the inner portion of the lower tarsal plate. This approach was applied as an adjunct to transconjunctival lower blepharoplasty. This case series presents the surgical method and outcomes in three patients, highlighting its potential role in improving lower lid contour and reducing postoperative complications.

## 2 Case History/Examination

**FIGURE 1:**
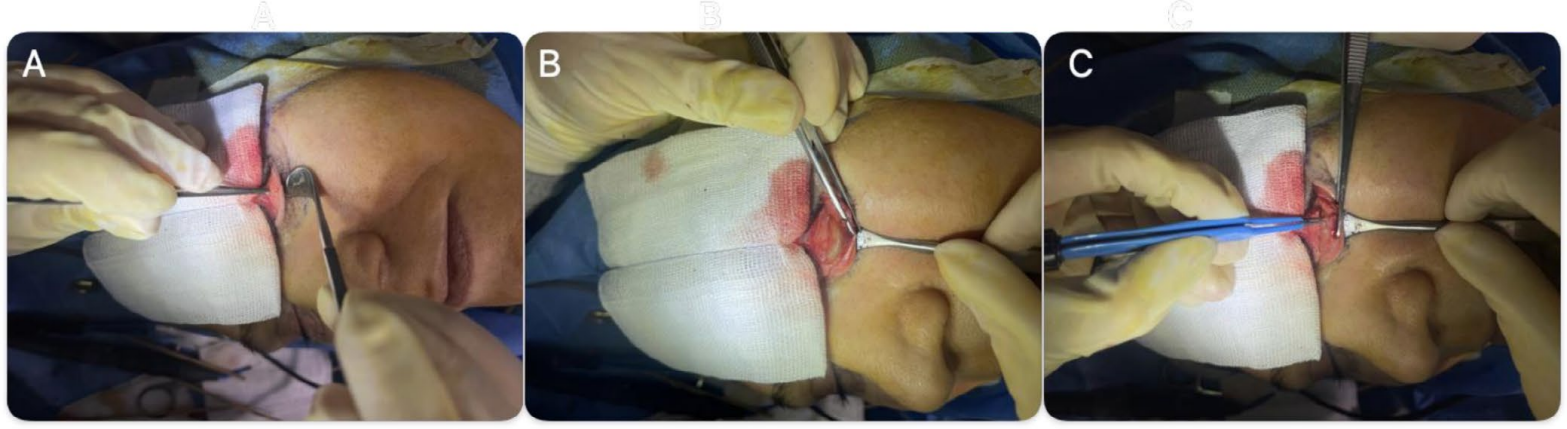
Demonstration of the electrocautery‐assisted internal lid‐tightening technique. (A) Eversion of the lower eyelid using a retractor. (B) Stabilization of the eyelid for precise application. (C) Low‐power coagulation applied at 5–6 points along the inner tarsal conjunctiva.

We present three patients who underwent lower eyelid blepharoplasty using a novel internal lid‐tightening technique. All patients demonstrated lower lid dermatochalasis with fat prolapse and mild eyelid laxity. Each procedure involved a transconjunctival incision with removal of herniated fat, followed by an adjunctive electrocautery maneuver applied to the inner surface of the lower tarsal plate (Figure 1). None of the patients had a history of prior eyelid surgery, severe dry eye, or systemic disease. Antiplatelet or anticoagulant medications, including aspirin, were discontinued 1 week before surgery.

**FIGURE 2:**
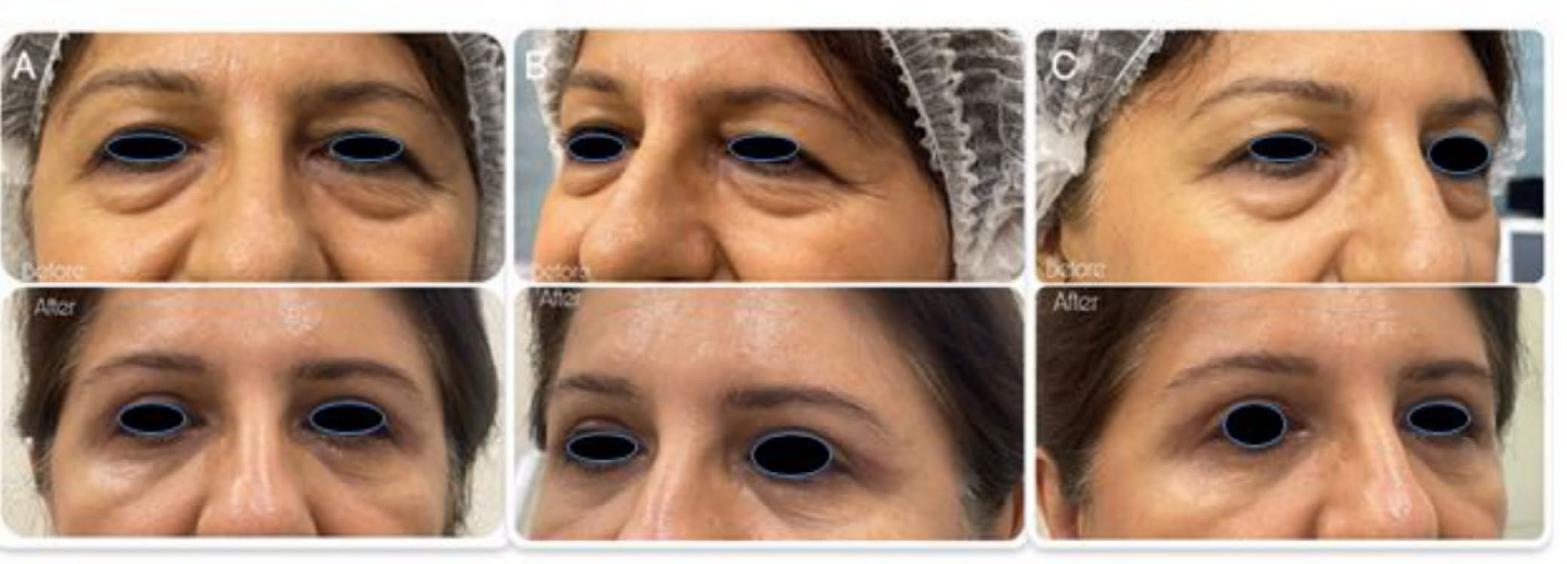
Preoperative and 1‐month postoperative photographs of the patient 1 (A: frontal view; B and C: lateral views) following lower blepharoplasty with the novel internal electrocautery technique.

Case 1 A 45‐year‐old woman presented with puffiness and skin laxity of the lower eyelids. She was otherwise healthy, with no history of ocular or cosmetic procedures. Examination showed normal lid laxity and a negative snap‐back test (Figure 2).

**FIGURE 3:**
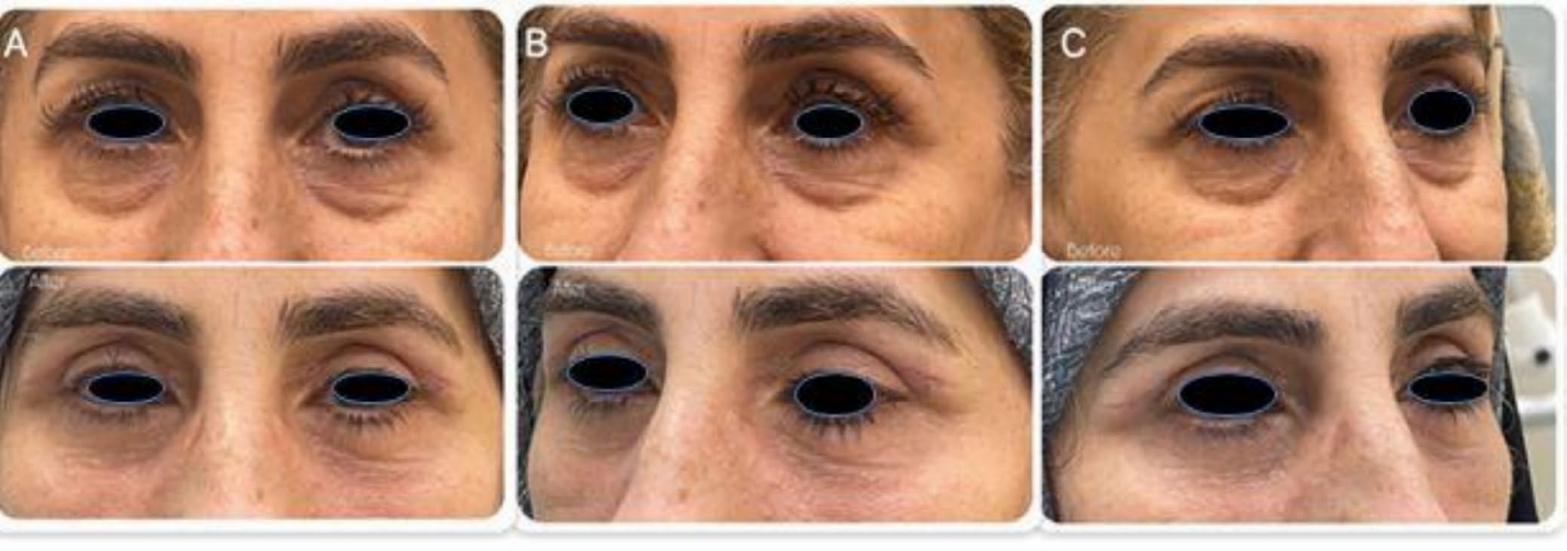
Preoperative and 1‐month postoperative photographs of Patient 2 (A: frontal view; B and C: lateral views) following lower blepharoplasty with the novel internal electrocautery technique. Simultaneous upper blepharoplasty was performed at the patient's request during the same surgical session.

Case 2 A 54‐year‐old woman presented with concerns of “bags” and mild looseness in the lower eyelids. She had no prior surgeries or dry eye symptoms, and a positive midface vector. Examination showed mild lid laxity (Figure 3).

**FIGURE 4:**
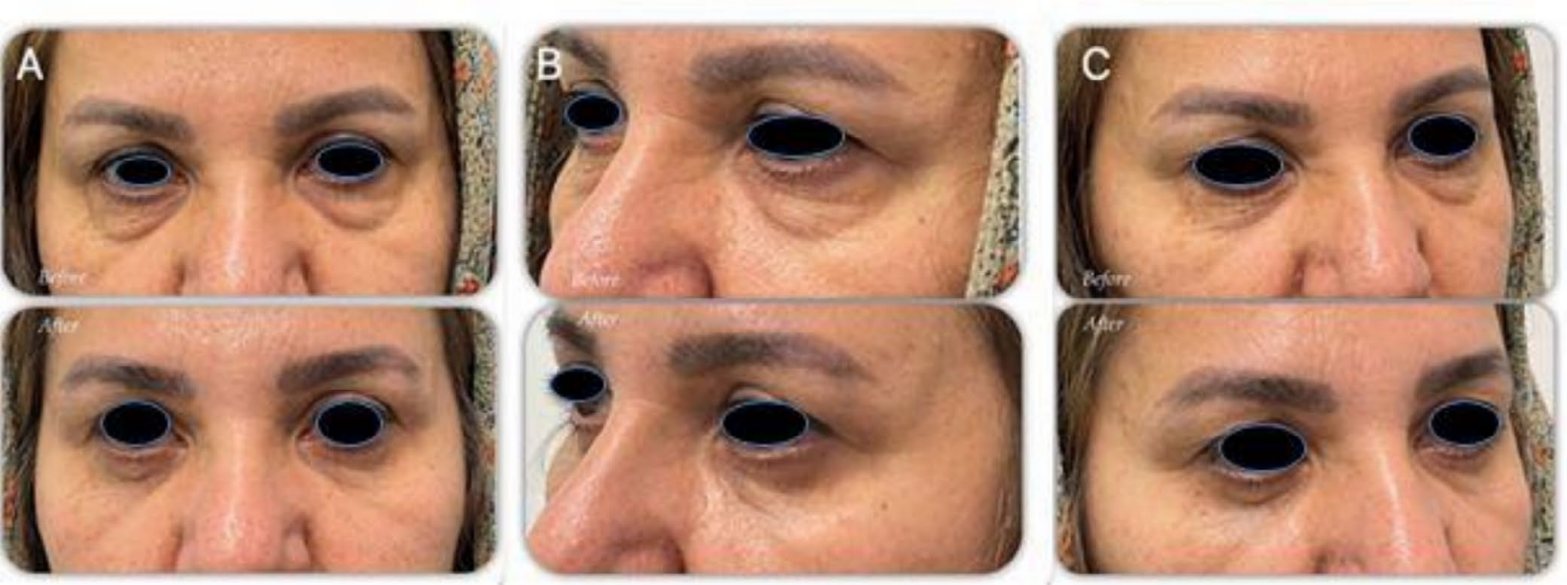
Preoperative and 1‐month postoperative photographs of Patient 3 (A: frontal view; B and C: lateral views) following lower blepharoplasty using the internal electrocautery‐assisted lid‐tightening technique.

Case 3 A 48‐year‐old woman presented with lower eyelid puffiness and fat prolapse. She had no systemic disease, ocular surgery, or dry eye history. Examination revealed moderate orbital fat herniation and normal canthal tendon tone (Figure 4).

## 3 Methods

All patients underwent transconjunctival lower blepharoplasty under local anesthesia (4% Citanest Plain). After thorough antiseptic preparation and draping, a transconjunctival incision was made on the inside of the lower eyelid, without any external skin marking or cut. This incision is typically placed just below the tarsal plate (about 2–4 mm below the lower lid margin) and extends nearly the width of the lid, allowing access to the orbital fat compartments without any visible external scar. Through this internal incision, the surgeon gently dissected to expose the herniated orbital fat pads in the lower eyelid. The protruding fat pockets (medial, central, and/or lateral pads) were carefully excised using electrocautery in the coagulation mode, which simultaneously cuts and cauterizes to minimize bleeding. Small bites of fat were removed until a smooth contour of the lower lid was achieved, using bipolar cautery as needed for hemostasis. This transconjunctival approach avoids disrupting the external skin and muscle and thus significantly reduces the risk of postoperative eyelid retraction compared to subciliary (transcutaneous) incisions. Once adequate fat removal was confirmed, achieving a flatter lower lid profile, attention was turned to enhancing eyelid support via a novel internal tightening maneuver.

With fat excision complete, the lower lid margin was gently everted using a small Desmarres retractor to expose the palpebral conjunctiva and the underlying tarsal plate from the inside. At this stage, the surgeon applied electrocautery at low power (approximately 8 W, coagulation mode) in a linear pattern directly to the inner surface of the lower tarsal plate, just beneath the lash line (as illustrated in Figure 1). Five to six evenly spaced cautery marks were made along the length of the tarsus. Each cautery application was superficial and targeted—just enough to create a small, controlled area of thermal injury within the tarsal tissue. The intent of these spaced cautery burns is to induce localized fibrosis (scar tissue formation) within the tarsal plate (the stiff supporting structure of the eyelid). As these cautery sites heal, the fibrosis contracts slightly, effectively tightening the posterior lamella of the eyelid. This internal scarring leads to a modest shortening and stiffening of the tarsus, which in turn enhances lower eyelid stability and resists outward sagging.

After completing the series of cautery applications, the transconjunctival incision was left unsutured. The small conjunctival incision inherently has excellent blood supply and tends to self‐seal rapidly; leaving it open allows any residual fluid to drain and avoids undue tension.

**FIGURE 5:**
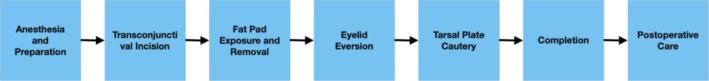
Illustration of Step‐by‐Step Operative Steps.

The step‐by‐step approach is illustrated in Figure 5.

Postoperative management included topical antibiotic and corticosteroid ointment (gentamicin and betamethasone) administered twice daily for 1 week, in addition to a single intramuscular injection of dexamethasone.

## 4 Results

All three patients tolerated the procedure well, and no intraoperative complications were observed. Postoperative pain, edema, and ecchymosis were mild and subsided spontaneously within 2 weeks. The transconjunctival approach effectively avoided external scarring, and none of the patients experienced ectropion, lid retraction, or conjunctival irritation. Importantly, the adjunctive internal tarsal electrocautery maneuver provided additional lower lid stability without adverse effects.

Case 1 At the 1‐month follow‐up, the patient demonstrated complete resolution of lower lid puffiness and stable lid contour with high satisfaction (Figure 2).

Case 2 Mild swelling and bruising subsided within 2 weeks, and by 1 month, the eyelid contours were symmetric and stable, with no retraction or asymmetry (Figure 3).

Case 3 At the 1‐month follow‐up, the lower eyelid contour was significantly improved with no complications, and lid tone was enhanced without requiring additional support procedures (Figure 4).

## 5 Discussion

Lower eyelid blepharoplasty remains a cornerstone of aesthetic eyelid surgery, particularly for correction of fat prolapse, dermatochalasis, and lid laxity. Among available approaches, the transconjunctival route has gained popularity due to its lower risk of visible scarring and postoperative malposition compared with transcutaneous (skin‐cutting) techniques [1, 3]. For example, Bhattacharjee et al. reported that transconjunctival blepharoplasty is associated with fewer cases of ectropion and skin‐related complications compared with transcutaneous approaches [7]. A recent systematic review of lower eyelid blepharoplasties confirms that, overall, lower lid blepharoplasty is a safe procedure with a low complication profile across various techniques [8].

Nevertheless, even with transconjunctival techniques, when lid laxity or borderline canthal support is present, adjunctive stabilizing procedures are often recommended. Traditional practice for patients with any appreciable eyelid looseness is to perform a canthal tightening procedure at the same time as the blepharoplasty [9]. This can be a lateral canthopexy (a minor tightening of the existing tendon) or a canthoplasty (a more invasive tendon shortening or reattachment) [10]. Tepper et al. 2015 reviewed 146 lateral canthal procedures performed alongside blepharoplasty and demonstrated ~86% anatomical success and ~90% patient satisfaction [11]. Similarly, Favarin et al. 2023 showed that routine inclusion of canthopexy in blepharoplasty resulted in a low rate (~2.94%) of eyelid malposition [4]. Hu et al. also described a “three‐step supporting technique” (septum tightening, orbicularis canthopexy, and muscle plication) in 697 patients, reporting > 94% nearly normal eyelid position with minimal complications, underscoring the protective role of adjunctive support in transcutaneous blepharoplasty [12].

Insights from studies on cauterization and conjunctival tissue contraction are also relevant. Superficial cauterization of the inferior bulbar conjunctiva in patients with moderate conjunctivochalasis demonstrated tissue contraction with minimal complications and no scarring, suggesting that controlled thermal injury can achieve clinically meaningful tightening effects [13]. In an animal model, Kim et al. compared electrocauterization with excision and suturing of conjunctival tissue, finding smoother healing and less scarring in cauterized eyes, thereby supporting the safety of carefully applied electrocautery for tissue stabilization [14].

In the present technique, instead of using sutures or canthal tendon modification, the series of internal tarsal cautery marks is intended to provide an *internal* support by stiffening the tarsus. This is akin to creating a mild cicatricial (scar) canthopexy effect without an external incision. The heat‐induced fibrosis in the tarsal plate augments the posterior lamella support, which can help prevent the lid from drooping or everting outward after surgery. It addresses the posterior lamella laxity directly, whereas a traditional canthopexy addresses horizontal laxity at the lid corner. The described method thus offers a novel, minimally invasive way to support the lower lid from inside.

The internal tarsal cautery technique described here can be seen as an innovative adjunct to the transconjunctival approach. Traditional transconjunctival blepharoplasty addresses fat prolapse but not lid laxity; if laxity is present, many surgeons would perform a separate canthopexy [15]. By performing the series of cautery spots on the tarsal plate, the surgeon induces mild shrinkage of the posterior lamella, which tightens the lid without any external incisions or implants. This is somewhat analogous to procedures in ophthalmology where cicatricial changes are induced to correct eyelid malpositions (e.g., cautery or scarification techniques for entropion or ectropion in certain cases), leveraging scar tissue to alter lid tension. While this internal cautery technique is not yet widely described in literature, it follows sound principles, creating a controlled scar to bolster a tissue's stiffness.

It is important to compare the efficacy and safety of this method with the more established canthal tightening sutures. A simple lateral canthopexy typically involves placing a suture from the lateral canthal tendon (or inferior crus) to the orbital rim periosteum to snug up the lid—a quick maneuver that has low morbidity and usually minimal effect on eye shape [15]. Studies have shown that such canthopexies can effectively correct mild‐to‐moderate laxity and drastically lower the incidence of postoperative lid sagging, without the downtime of a formal canthoplasty [16]. The cautery method achieves tightening without a suture, which eliminates any risk of suture‐related complications or visible knot. However, one must ensure the cautery is precisely controlled; overly aggressive cautery could theoretically cause full‐thickness tarsal damage or contour irregularities on the eyelid. In the described series, using low‐power settings and a limited number of spots avoided any such issues, and the conjunctiva healed without incidents. Patients benefited by having improved lower lid tone, and no cases of postoperative ectropion were observed (consistent with the low malposition rates expected from a transconjunctival approach).

In summary, our case series builds on these findings by adapting low‐power electrocautery applied to the inner surface of the lower tarsal plate during transconjunctival blepharoplasty. Rather than relying solely on conjunctival shrinkage, the maneuver targets the tarsal plane after fat removal, thereby tightening the posterior lamella while preserving external tissue integrity. The favorable outcomes observed—including minimal swelling, absence of ectropion or lid retraction at 1‐month follow‐up, and improved contour stability—are consistent with prior reports demonstrating the benefits of reinforcing internal support structures while avoiding external incisions or sutures.

However, limitations must be acknowledged. The long‐term durability of this tarsal cautery‐based tightening has yet to be established as most reports of conjunctival cauterization are limited to 6–12 months of follow‐up. Risks such as over‐shrinkage, scarring, or conjunctival irritation remain possible if energy delivery is not carefully controlled. Objective measurements of eyelid position, such as distraction distance, scleral show, or canthal tilt, were not recorded and should be incorporated in future studies. Larger comparative studies against standard adjunctive procedures (e.g., lateral canthopexy) are warranted to define optimal patient selection criteria and confirm long‐term safety.

## Author Contributions

**Afsaneh Sadeghzadeh Bazargan:** project administration, writing – original draft. **Alireza Jafarzadeh:** conceptualization, investigation, methodology, project administration, writing – original draft, writing – review and editing. **Roya Zeinali:** data curation, writing – original draft.

## Funding

The authors have nothing to report.

## Disclosure

**Transparency Declaration:** The authors affirm that the manuscript is honest, accurate, and transparent. No important aspect of the study has been omitted.

## Ethics Statement

Written informed consent was obtained from all patients, and their privacy was maintained in accordance with ethical standards. All patient photos included in this manuscript have been anonymized to ensure patient confidentiality. Based on the guidelines of the local medical research ethics committee, registration of this study is not required.

## Conflicts of Interest

The authors declare no conflicts of interest.

## Data Availability

The data that support the findings of this study are available from the corresponding author upon reasonable request.
